# Generation of mice harbouring a conditional loss-of-function allele of *Gata6*

**DOI:** 10.1186/1471-213X-6-19

**Published:** 2006-04-12

**Authors:** Chhinder P Sodhi, Jixuan Li, Stephen A Duncan

**Affiliations:** 1 Department of Cell Biology, Neurobiology & Anatomy, Medical College of Wisconsin, Milwaukee, WI, USA

## Abstract

The zinc finger transcription factor GATA6 is believed to have important roles in the development of several organs including the liver, gastrointestinal tract and heart. However, analyses of the contribution of GATA6 toward organogenesis have been hampered because *Gata6*^-/- ^mice fail to develop beyond gastrulation due to defects in extraembryonic endoderm function. We have therefore generated a mouse line harbouring a conditional loss-of-function allele of *Gata6 *using *Cre*/*lox*P technology.

*LoxP *elements were introduced into introns flanking exon 2 of the *Gata6 *gene by homologous recombination in ES cells. Mice containing this altered allele were bred to homozygosity and were found to be viable and fertile. To assess the functional integrity of the *loxP *sites and to confirm that we had generated a *Gata6 *loss-of-function allele, we bred *Gata6 *'floxed' mice to *EIIa-Cre *mice in which Cre is ubiquitously expressed, and to *Villin-Cre *mice that express Cre in the epithelial cells of the intestine. We conclude that we have generated a line of mice in which GATA6 activity can be ablated in a cell type specific manner by expression of Cre recombinase. This line of mice can be used to establish the role of GATA6 in regulating embryonic development and various aspects of mammalian physiology.

## Background

The mouse *Gata6 *gene encodes a 45 kD protein containing two highly conserved zinc-finger DNA binding domains with a Cys-X_2_-Cys-X_17_-Cys-X_2_-Cys motif that directs binding to the nucleotide sequence element (A/T)GATA(A/G) 1. GATA4, 5 and 6 make up a subset of GATA factors that have been implicated in the development of several organs including the heart, lung, gastrointestinal tract and liver [1-4]. Development of GATA6 null embryos arrests during gastrulation as a consequence of defects in extraembryonic endoderm function [2,5]. This early embryonic lethality can be rescued by complementing the GATA6 null embryos with a wild type extraembryonic visceral endoderm using tetraploid embryo complementation, and embryos derived by this process can survive until E10.5 [3]. Analyses of such *Gata6*^-/-^ES cell-derived embryos has revealed defects in hepatogenesis, which supports the proposal that GATA6 is an important developmental regulator. Although the ability to generate embryos from *Gata6*^-/-^ES cells by tetraploid embryo complementation has provided important insight into the contribution of GATA6 during early embryogenesis, this approach is not compatible with studying the role of GATA6 at later stages in development or its role in controlling differentiation of specific cell types. In the current report, we describe the generation of mice containing a conditional null allele of *Gata6 *that can be used for cell type-specific removal of GATA6 by Cre-mediated recombination.

## Results and discussion

**Figure 1:**
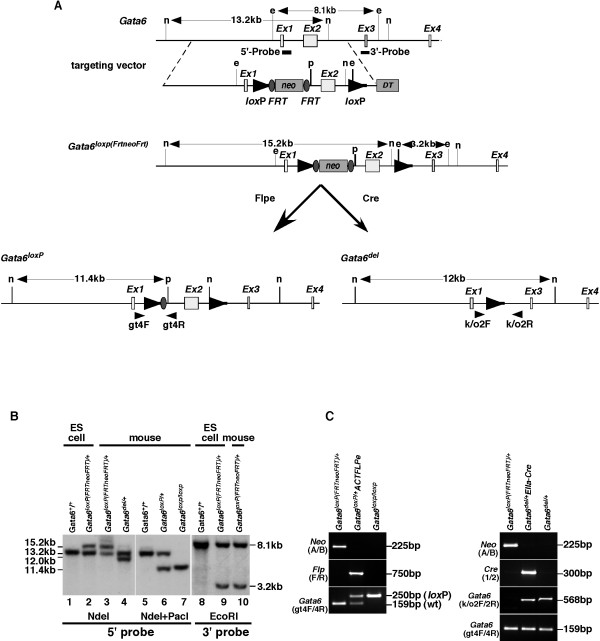
**Generation of a *Gata6 conditional null *allele**. (A) Schematic showing a map of the *Gata6 *genomic locus and the targeting vector with exons represented by open boxes. The relative position of Southern blot probes (lines), PCR primers (small arrowheads), *loxP *(large arrowheads) and FRT (ovals) sites, as well as cassettes encoding neomycin phosphotransferase (neo) and diphtheria toxin (DT), are included. Sizes of relevant *Eco*RI (e), *Nde*I (n), and *Pac*I (p) restriction endonuclease fragments are shown in kilobase pairs (kb). (B) Southern blot analysis of genomic DNA isolated from ES cells (lanes 1, 2, 8 and 9) or mouse tails (lanes 3–7 and 10). An example of an ES cell line containing a correctly targeted *Gata6*^*loxP(FRTneoFRT) *^allele is shown in lanes 2 and 8. Mice harbouring the modified *Gata6 *allele were generated from these ES cells (lanes 3 and 10). Exon 2 of *Gata6 *was deleted (*Gata6*^*del*^) or the *neo *cassette alone was deleted, leaving *Gata6 *exon 2 flanked by *lox*P elements (*Gata6*^*loxP*^), by breeding *Gata6*^*loxP(FRTneoFRT) *^mice to transgenic mice expressing either Cre (lane 6 and 7) or Flp (lane 4) recombinases, respectively. The size of restriction fragments identified by 5' and 3' probes (Fig. 1A) was deduced from their position relative to standard DNA fragments. (C) The genotypes of mice and embryos were also determined by PCR amplification of genomic DNA. Primers were designed that differentiated between the *Gata6*^+ ^(gt4F/4R; 159 bp), *Gata*^*loxP *^(gt4F/4R; 250 bp) and *Gata6*^*del *^(k/o2F/2R; 568 bp) alleles, as well as *neo *(Neo A/B; 225 bp), *flp *(Flp F/R; 750 bp), and *cre *(Cre 1/2; 300 bp) transgenes.

To ensure the elimination of GATA6 activity, we chose to flank *Gata6 *exon 2 (based on nomenclature described by Brewer *et al *[6,7]) with *lox*P elements because this exon encodes the majority of the GATA6 protein (Fig. 1A). We decided to use a recombineering approach to facilitate the accurate placement of the *lox*P elements following the procedure described by Lee *et al *[8]. The final targeting vector contained a *lox*P element located between exons 2 and 3. In addition, a cassette containing a *lox*P element lying immediately 5' to a *neomycin phosphotransferase *(*neo*) gene, which conferred resistance to G418, was introduced between exons 1 and 2 (Fig. 1A). FRT sites flanked the *neo *gene, in order to allow removal of *neo *by Flp recombinase. Two novel restriction endonuclease cleavage sites (*Eco*RI and *Pac*I) were also introduced into the *Gata6 *targeting vector to allow the identification of correctly modified *Gata6 *alleles by Southern blot analyses. The targeting vector was introduced into R1 ES cells [9] by electroporation, and G418-resistant ES cell clones were tested for homologous recombination at the *Gata6 *locus by Southern blot analysis. Fig. 1B shows an example of a correctly targeted ES (*Gata6*^*loxP*(*FRTneoFRT*)/+^) cell line. Following digestion of ES cell genomic DNA with *Eco*RI, a probe that lies 3' to the short arm of homology used in the targeting vector identified an expected 8.1 kb wild type *Gata6 *DNA fragment, while correctly targeted cells contain an additional 3.2 kb fragment due to the introduction of a new *Eco*RI site. When *Nde*I digested DNA was probed with a DNA fragment lying 5' to the expected position of the introduced *lox*P site, it identified the predicted 13.2 kb wild type fragment and a novel 15.2 kb fragment, which resulted from introducing the *neo *cassette into the *Gata6 *locus. Five correctly targeted ES cell clones were recovered and three of these cell lines were used to generate chimeric mice by morula aggregation [10]. These chimeric animals were then mated with CD-1 mice and successful germline transmission of the *Gata6*^*loxP*(*FRTneoFRT*) ^allele was confirmed by Southern blot analysis of tail DNA (Fig. 1B). Mice carrying a single *Gata6*^*loxP*(*FRTneoFRT*)/+ ^allele were viable and fertile. However, we were unable to obtain mice that were homozygous for this allele, which suggested that the inclusion of the *neo *cassette disrupted GATA6 function and resulted in embryonic lethality as observed for *Gata6*^-/- ^embryos [2,5]. We therefore established a mouse line, *Gata6*^*loxP*/+^, that lacked the *neo *cassette by inducing recombination between the *FRT *sites *in vivo *[11]. This was achieved by mating *Gata6*^*loxP*(*FRTneoFRT*)/+ ^mice to a transgenic mouse, B6;SJL-Tg(ACTFLPe)9205Dym/J, in which Flp recombinase is widely expressed from the human *beta actin *gene promoter [11]. Correct excision of the *neo *cassette was confirmed in offspring by Southern blot and PCR analyses (Fig. 1B & C). *Gata6*^*loxP*/+ ^mice were finally interbred to generate *Gata6*^*loxP*/*loxP*^mice, which are healthy, fertile and have reached maturity.

**Figure 2:**
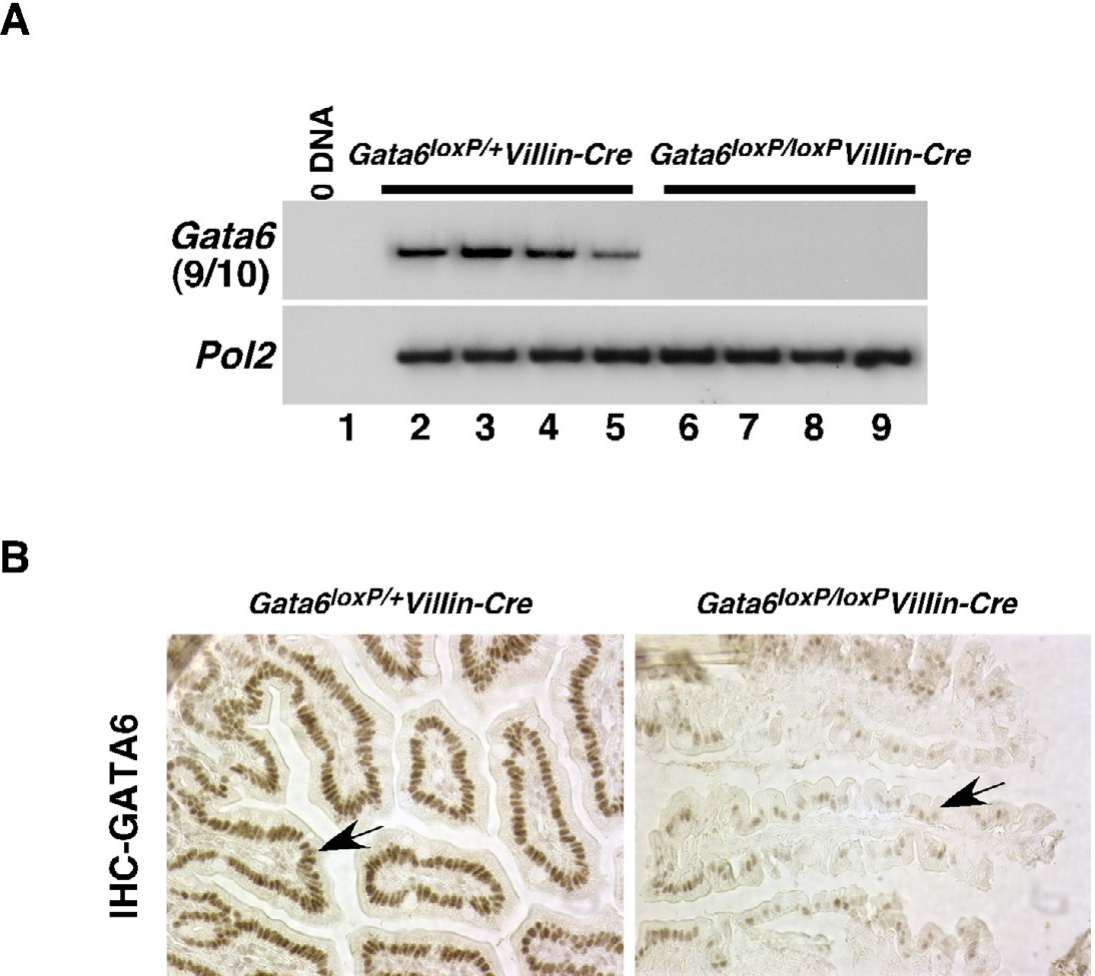
***Gata6 *can be successfully ablated in the intestine of *Gata6*^*loxP*/*loxP *^mice by expression of Cre from the *Villin *promoter**. (A) The steady-state level of *Gata6 *mRNA was compared in intestines isolated from four control *Gata6*^*loxP*/+^*Villin-Cre *(lanes 2–5) or experimental *Gata6*^*loxP*/*loxP*^*Villin-Cre *(lanes 6–9) mice. Amplification of Pol2 (*Polr2a*) mRNA showed that similar levels of starting material were utilized in each reaction. (B) Immunohistochemistry showing that GATA6 was detected as an abundant nuclear protein in epithelial cells (arrows) of control intestines, but was detected at greatly reduced levels in *Gata6*^*loxP*/*loxP*^*Villin-Cre *intestines.

We next addressed whether removal of GATA6 could be induced *in vivo *by expression of Cre recombinase. We chose to disrupt the *Gata6 *gene in the gastrointestinal tract because it is highly expressed in the epithelium and may have roles in controlling gut physiology or development. To produce *Gata6*^*loxP*/+^*Villin-Cre *mice, we bred *Gata6*^*loxP*/*loxP *^mice with transgenic mice (Tg(Vil-cre)997Gum) in which Cre expression was directed to the epithelial cells of the small intestine by the *Villin *(*Vil1*) promoter [12]. Using this transgenic strain of mice, Cre activity can be detected in the epithelium of the small intestine from E14.5 [12]. *Gata6*^*loxP*/+^*Villin-Cre *males were mated with *Gata6*^*loxP*/*loxP *^females to obtain *Gata6*^*loxP*/+^*Villin-Cre *control and *Gata6*^*loxP*/*loxP*^*Villin-Cre *experimental offspring. In previous experiments, we observed that the efficiency through which Cre mediates recombination between *lox*P sites varies between different transgenic male mice [13]. We noted a similar situation when using various *Gata6*^*loxP*/+^*Villin-Cre *males. In the majority of cases (4/5 stud males) we were unable to obtain *Gata6*^*loxP*/*loxP*^*Villin-Cre *offspring (n = 95 mice genotyped), and this lethality associated with presumptive loss of GATA6 in the intestine is currently under investigation. However, one *Gata6*^*loxP*/+^*Villin-Cre *male did produce offspring (12/90) whose genotype was determined to be *Gata6*^*loxP*/*loxP*^*Villin-Cre *by PCR analysis of tail DNA. We, therefore, used RT-PCR with *Gata6 *primers corresponding to nucleotide sequences predicted to be deleted after recombination between *Gata6 lox*P elements, to compare the steady-state levels of *Gata6 *mRNA in intestines isolated from control and experimental mice generated by this particular *Gata6*^*loxP*/+^*Villin-Cre *male. Fig. 2A shows that *Gata6 *mRNA could be detected in control intestine but not in intestine isolated from a subset of *Gata6*^*loxP*/*loxP*^*Villin-Cre *offspring. The remaining *Gata6*^*loxP*/*loxP*^*Villin-Cre *mice were found to continue to express *Gata6 *mRNA at varying levels (data not shown). We also examined expression of GATA6 protein in the intestines of *Gata6*^*loxP*/*loxP*^*Villin-Cre *offspring derived from the same Gata6^*loxP*/+^*Villin-Cre *male. Fig. 2B shows that GATA6 was detected as an abundant nuclear protein in the epithelial cells of control intestines. However, *Gata6*^*loxP*/*loxP *^*Villin-Cre *mice displayed a marked reduction in the abundance of intestinal GATA6 (Fig. 2B). From these results, we conclude that the *Gata6 *gene can be conditionally disrupted in *Gata6*^*loxP*/*loxP *^mice by cell-type specific expression of Cre recombinase.

Cre-mediated deletion of exon2 in *Gata6*^*loxP*/*loxP *^mice was predicted to result in loss of GATA6 function. If this were true, we expected that embryos homozygous for the deleted allele (*Gata6*^*del*^) should undergo developmental arrest, as was described for *Gata6*^-/- ^embryos [2,5]. *Gata6*^*del*/+ ^mice were produced by mating *Gata6*^*loxP*(*FRTneoFRT*)/+ ^mice with an *EIIa-Cre *transgenic mouse (B6.FVB-Tg(EIIa-cre)C5379Lmgd/J). The *EIIa-Cre *mouse expresses Cre recombinase in nearly all tissues, including those of pre-implantation embryos, and has been used previously to mediate recombination between *lox*P sites in germ cells [14]. *Gata6*^+/*del *^progeny were identified by Southern blot analyses of *Nde*I digested tail DNA. As shown in Fig. 1B, Southern blots hybridized with the 5' *Gata6 *probe (Fig. 1A) revealed the conversion of a 15.2 kb *Nde*I fragment from the *Gata6*^*loxP*(*FRTneoFRT*) ^allele to an expected 12 kb *Nde*I fragment from the *Gata6*^*del *^allele. *Gata6*^+/*del *^were then mated *inter se*, and the genotype of embryos collected between E8.5 and E11.5 was determined by PCR analysis of genomic DNA. Of 77 embryos recovered, 27 were *Gata6*^+/+ ^and 50 were *Gata6*^+/*del *^(Table 1). In addition, 23 partially resorbed empty decidual masses were recovered, which we believe likely resulted from the early developmental arrest of *Gata6*^*del*/*del *^embryos. We finally determined whether the developmental lethality associated with loss of GATA6 could be induced by expression of Cre in pre-implantation embryos. To achieve this, *Gata6*^*loxP*/*loxP *^mice were bred to *Gata6*^+/*del*^*EIIa-Cre *mice, which are heterozygous for the *EIIa-Cre *transgene. The genotype of resulting embryos ranging from E8.5 to E11.5 was again determined by PCR. While we recovered 14 *Gata6*^*loxP*/+^, 18 *Gata6*^*loxP*/*del*^, and 16 *Gata6*^*loxP*/+^*EIIaCre *embryos, no *Gata6*^*loxP*/*del*^*EIIaCre *embryos were identified although 13 empty resorbing decidual masses were observed (Table 1).

**Table 1:** Embryonic lethality associated with loss of GATA6 function

*Gata6*^+/*del *^X *Gata6*^+/*del*^		*Gata6*^*loxP*/*loxP *^X *Gata6*^+/*del *^*EIIa-Cre*	
genotype	E8.5 – 11.5 embryos	genotype	E8.5 – 11.5 embryos

*+/+*	27	*loxP/+*	14
*+/del*	50	*loxP/+ EIIa-Cre*	16
*del/del*	0	*loxP/del*	18
resorbed	23	*loxP/del EIIa-Cre*	0
		resorbed	13

## Conclusion

In summary, we conclude that we have generated a line of mice in which GATA6 transcriptional activity can be ablated by expression of Cre. We believe that the availability of this mouse will be useful to elucidate the contribution of GATA6 during organogenesis as well as its physiological role in adult mice.

## Methods

### Plasmid construction

A bacterial artificial chromosome (BAC # RP23-410I12) that contained *Gata6 *genomic DNA was obtained from BACPAC Resources, Children Hospital Research Institute, Oakland, CA. Plasmid pNeb-DT-GATA6 was generated by cloning a 12.2 kb EcoRV/PacI genomic fragment containing *Gata6 *exons 1 and 2 into the PacI/HincII site of a vector, pNEB193-DT, which contained a diphtheria toxin (DT) expression cassette to enrich against random integration of the targeting plasmid in ES cells. A cassette containing *loxP-neo-loxP *was amplified by PCR from the plasmid pSV-LNL (modified from Zhang *et al*)[15] using the following primers: *Gata6ET1:*GCTTGCTGTTTGAGTCTACCCCATTTCTGCCTGTTTCTTGACATCCCTTCGAATTCTGGTACCGGCGCGCCTAGTCGAC, *Gata6ET2:*ATCCATTATTGTCAATGTCTAAAGATGGAATTGTCTCTGCACAAGCTATCTTCTCAACTCGAGCCCTTAATTAACCGGT. These oligonucleotides contained 55 bp of sequence from *Gata6 *intron 2 (underlined). This amplicon was introduced into *Gata6 *intron 2 sequence in pNEB-DT-GATA6 by homologous recombination in *E.coli *following the procedure described by *Lee et al *[8] The *loxPneoloxP *cassette was then converted to a single *lox*P site by expression of Cre recombinase [8]. A *loxP(FRTneoFRT) *cassette was then amplified from pSV-LFNF using primers *Gata6ET3:*CACGCTGGTGGTTGTAAGGCGGTTTGTGTTTAAGGTGTGCGGTTGGCCTGGACGTGTGGTACCGGCGCGCCTAGTCGAC, *Gata6ET4:*GAAAAAGTTACCTAGCCCAGAGAAAGTGAGATGCCAGGAAAGGCATAAGGATATCAACTCGAGCCCTTAATTAACCGGT. These oligonucleotides contain 56 bp (Gata6ET3) and 52 bp (Gata6ET4) of sequence from *Gata6 *intron 1, respectively. This cassette was introduced into *Gata6 *intron 1, again using homologous recombination in *E.coli*. to generate the final targeting vector (Fig. 1A).

### ES cell targeting and animals

Linear targeting vector (100μg) was introduced into R1 ES cells by electroporation, and the genotype of colonies resistant to 350μg/ml of Geneticin (Gibco BRL) was determined by Southern blot (Fig. 1B). Chimeric mice were generated by aggregation of ES cells with CD-1 morulae as described previously [10] and the modified allele was passed through the germline by breeding chimeras to CD1 mice. *Gata6*^*loxP*/*loxP *^mice were produced by breeding *Gata6*^*loxp*(*FRTneoFRT*)/+ ^mice to B6;SJL-Tg(ACTFLPe)9205Dym/J mice [16] (Jackson Labs) to delete the *FRTneoFRT *cassette by Flp-mediated recombination in the germline. The *ACTFLPe *transgene was removed by breeding F_1 _*Gata6*^*loxP*/*loxP *^mice into CD-1 mice. *Gata6*^+/*del *^mice were generated by mating *Gata6*^*loxp*(*FRTneoFRT*)^/^+ ^animals with B6.FVB-Tg(EIIa-cre)C5379Lmgd/J transgenic mice [17] (Jackson Labs) to allow Cre-mediate recombination between *lox*P elements in the germline. The *EIIa-Cre *transgene was removed by breeding *Gata6*^+/*del *^F_1 _mice with CD1 mice. The MCW IACUC committee approved all procedures using animals.

### Southern blot, PCR and RT-PCR

Southern blot analyses were performed using standard conditions with probes indicated in Fig. 1. Genotypes were determined by PCR using the following oligonucleotide primer pairs: *Gata6 gt4F*/4R, GTGGTTGTAAGGCGGTTTGT, ACGCGAGCTCCAGAAAAAGT; *Gata6 k/o*2F/2R, AGTCTCCCTGTCATTCTTCCTGCTC, TGATCAAACCTGGGTCTACACTCCTA; *Flp F/R*, GGTCCAACTGCAGCCCAAGCTTCC, GTGGATCGATCCTACCCCTTGCG [16]; *Cre 1/2*, GTTCGCAAGAACCTGATGGACA, CTAGAGCCTGTTTTGCACGTTC [18]; *Neo A/B*, GCCAACGCTATGTCCTGATAGCGGT, AGCCGGTCTTGTCGATCAGGATGAT. RT-PCR was performed as described previously [19] with the following primer pairs: *Gata6 9/10; *AGTTTTCCGGCAGAGCAGTA, AGTCAAGGCCATCCACTGTC, *Pol2 F/R; *CTGATGCGGGTGCTGAGTGAGAAGG, GCGGTTGACCCCATGACGAGTG.

## Immunohistochemistry

Immunohistochemistry was performed using antigen retrieval in citrate buffer as described previously [13] using an anti-GATA6 antibody (AF1700 R&D Systems,1/1000 dilution).

## Abbreviations

Neo, neomycin phosphotransferase

## Authors' contributions

C.P.S. generated the targeting vector and carried out analyses of conditional knockout mice, as well as contributed to experimental design and draft of the manuscript. J. L. generated aggregation chimeras. S.A.D. conceived of the study, contributed to experimental design and interpretation of results, and coordinated the project and writing of the manuscript.
